# Personal

**Published:** 1898-12

**Authors:** 


### PERSONAL.

Dr. A. H. Briggs, late Major-surgeon of the Sixty-fifth Regiment, and Captain E. L. Ruffner and Harry A. Mead, assistant surgeons, have been mustered out of the United States service, and have resumed the practice of medicine. Dr. Briggs's office and residence is at 267 Hudson street; Dr. Mead's at 758 Elmwood avenue, and Dr. Ruffner's at 181 Allen street.

Dr. James T. Jelks, of Hot Springs, Ark., editor of the Hot Springs *Medical Journal*, and one of the most prominent physicians in the southwest, recently spent a few days in Buffalo and Niagara Falls. Dr. Jelks was on his way east to enjoy a vacation and a visit to the principal medical centers.

Dr. Grosvenor R. Trowbridge has established his office at Suite No. 416, Ellicott square. Hours, 11-1 and 2-4. Telephone, Seneca 268. His residence remains at 1331 Main street. Telephone, Bryant 2813.

Dr. Joseph D. Bryant, of New York, was elected president of the New York State Medical Association at its last meeting in that city. The association did itself much credit in this selection of a presiding officer.

Dr. T. M. Crowe, of Buffalo, has removed his offices from 703 Elk street to 847 Ellicott square. Hours, 9-10 a. m., 2-4 and 7-8 p. m. Day calls, telephone, Seneca 970. Night calls, Seneca 41.

Dr. George Gillett Thomas, of Wilmington, N. C., has been appointed Chief Surgeon of the Atlantic Coast Line railway system. Dr. Thomas is an accomplished surgeon, and his selection is a credit to the great railway that has chosen him.

---

Acting Assistant Surgeon Nelson W. Wilson, by a recent order of the War Department, is assigned to duty as examiner of recruits at Buffalo, in addition to his duties as assistant to the Surgeon at Fort Porter.

---

Majors C. Frank Bruso and Francis T. Metcalfe, of Buffalo, Brigade Surgeons, U. S. Volunteers, have been mustered out of the military service, their services being no longer required.

---

Dr. Thomas H. McKee, of Buffalo, U. of B. '98, has been appointed house surgeon at the Riverside Hospital.

---

Dr. Montgomery A. Crockett, of Buffalo, announces his resignation as gynecologist to the Riverside Hospital.

